# A Novel Cryptic Virus Isolated from *Galphimia* spp. in Mexico

**DOI:** 10.3390/pathogens13060504

**Published:** 2024-06-13

**Authors:** Dianella Iglesias, Kristian Stevens, Ashutosh Sharma, Alfredo Diaz-Lara

**Affiliations:** 1 School of Engineering and Sciences, Tecnologico de Monterrey, Campus Queretaro, Queretaro 76130, Mexico; diglesias@tec.mx; 2 Departments of Computer Science and Evolution and Ecology, University of California-Davis, Davis, CA 95616, USA; kastevens@ucdavis.edu

**Keywords:** cryptic virus, high-throughput sequencing, medicinal plant, Mexico

## Abstract

*Galphimia* spp. is a plant employed in traditional medicine in Mexico because of its anxiolytic and sedative effects. Viruses have been associated with different alterations in plants, although asymptomatic agents (i.e., cryptic viruses) are also known. High-throughput sequencing (HTS) allows for the detection of pathogenic and non-pathogenic viral agents in plants, including potential novel viruses. The aim of this study was to investigate the presence of viral agents in two populations of *Galphimia* spp. by HTS. Sequencing was conducted on an Illumina NextSeq 550 platform, and a putative novel virus was identified. Two contigs showed homology to partitiviruses, and these encoded the RNA-dependent RNA polymerase and coat protein. These proteins showed the highest identities with orthologs in the recently discovered Vitis cryptic virus. A phylogenetic analysis of both RNAs showed that the new virus clusters into the monophyletic genus *Deltapartitivirus* along with other plant-infecting viruses. The result of the HTS analysis was validated by conventional RT-PCR and Sanger sequencing. A novel virus was discovered in a symptomless *Galphimia* spp. plant and tentatively named the Galphimia cryptic virus (GCV). This is the first virus discovered in medicinal plants in Mexico.

## 1. Introduction

*Galphimia* spp. is a wild plant endemic to Mexico [1], that belongs to the Malpighiaceae family [2]. It is well known for its medicinal properties, among which anxiolytic and sedative properties stand out [3,4]. Several studies have been carried out on this plant to increase the production of metabolites with sedative activity [5,6,7]. Consequently, it is crucial to identify stressing factors (biotic and abiotic) that might affect the plant’s metabolism. Viruses have been associated with different alterations in plants, although asymptomatic agents (i.e., cryptic viruses) are also known [8]. During asymptomatic infections, viral replication generally does not occur, and viruses remain in a silent/active state. It generates a certain tolerance by the plant to the virus; thus, the virus titer is reduced to avoid cytopathic effects and visible damage [9]. In terms of the Malpighiaceae family, and *Galphimia* genus, no infections by pathogens, such as by a virus or a viroid have been reported.

High-throughput sequencing (HTS) allows for the detection of pathogenic and non-pathogenic viral agents in plants, including potential novel viruses [10]. With plant RNA sequencing (RNA-seq), it is possible to obtain the sequence information of viruses and viroids in plant tissues. Data from RNA-seq experiments combined with molecular tests (i.e., PCR) are effective for virome analysis [11]. During the detection of plant viruses via HTS, templates such as single-stranded RNA (ssRNA), total RNA, double-stranded RNA (dsRNA), or preparations enriched by virus-like particles have been used. With the widespread use of HTS technology, there has been a dramatic increase in the number of viruses discovered [12], making the virome of most plants an accessible object of study. 

Partitiviruses (family *Partitiviridae*) have small bi-segmented or tri-segmented dsRNA genomes, with each segment coding for a single gene, an RNA-dependent RNA polymerase (RdRp), and one or two putative coat proteins (CPs) [13]. Partitiviruses can infect fungi, plants, and protozoa [14]. Those that infect plants are classified specifically in the genera *Alphapartitivirus*, *Betapartitivirus,* and *Deltapartitivirus,* and they are called “cryptic viruses”. These are transmitted intracellularly via seeds through infection of the ovules or pollen [15]. Most of the studied partitiviruses are associated with latent infections [16]. The interaction between the virus and the host plant has been commonly classified as mutualism [13]; this can influence many processes, including plant metabolism. For all the above, our study aimed to investigate the presence of viral agents, including known and unknown viruses in two populations of *Galphimia* spp. by HTS.

## 2. Materials and Methods

### 2.1. Plant Material, RNA Extraction, and High-Throughput Sequencing

**Figure 1:**
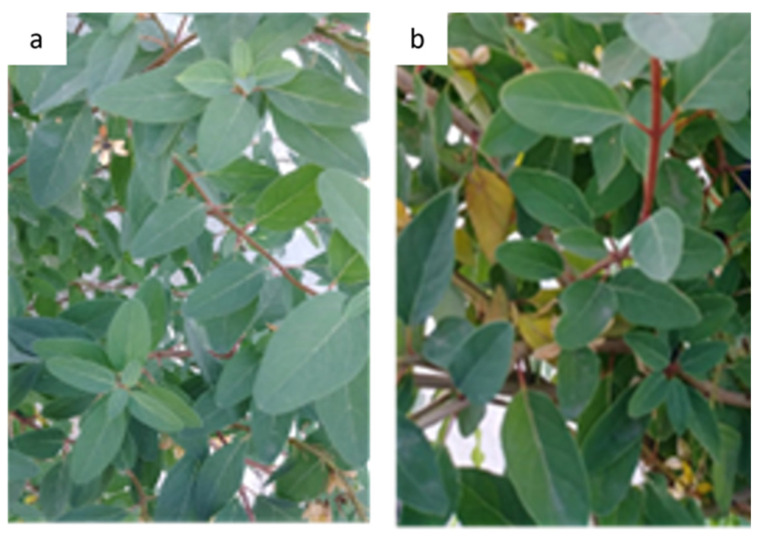
Asymptomatic leaves in *Galphimia* spp. plants. (**a**), Guanajuato population; (**b**), Morelos population.

Seeds of two populations of *Galphimia* spp. originally from Morelos and Guanajuato, Mexico, were germinated and grown under greenhouse conditions. Three samples were obtained, G2 and G3 of Guanajuato population and T2 of Morelos. Total RNA was extracted from asymptomatic leaves in both populations (Figure 1) using RNeasy Plant Mini Kit from QIAGEN. HTS libraries were prepared using the Illumina TruSeq Stranded Total RNA protocol. Sequencing was conducted on an Illumina NextSeq 550 platform using a paired-end 76 bp regime.

### 2.2. Data Processing and Phylogenetic Analysis 

De novo transcriptome assembly was carried out by rnaSPAdes (v. 3.15.4). To identify viral contigs, BLASTx analysis was performed against the GenBank non-redundant protein database, E-value: 1.0 × 10^−3^. To determine the phylogenetic relationship between the putative novel virus and previously reported viruses, a neighbor-joining tree was constructed using CLCBIO with 1000 bootstrap replicates based on the alignment of RdRp and CP amino acid sequences using MUSCLE (v.5). Only edges with greater than 50% bootstrap support were included in the tree.

### 2.3. Validation by RT-PCR

HTS findings were validated by two-step reverse transcription PCR (RT-PCR) on the original extraction. Employing primers that target two different virus genomic regions, RdRp and CP, the presence of the putative virus in the source plants (i.e., RNA extracts) was determined (Table 1). Such primers were designed by Primer3 plus program using default parameter settings.

Initially, 7 µL of RNA was mixed with 3 μL of random hexamer primers (0.1 µg/µL, working concentration). RNA was denatured in thermocycler by setting up for 3 min at 94 °C and then 7 min at 68 °C. Immediately, samples were put on ice. The first-strand cDNAs were synthesized from the working solution (10 μL) in a final 20 μL reaction volume employing Invitrogen 5X First strand buffer, 0.1 M DTT, 10 umol dNTPs mix, and Invitrogen Superscript II Rtase (200 U/µL). Samples were put in the thermocycler, and the following cycle was run: 25 °C for 15 min, 42 °C for 90 °C min, 72 °C for 15 min, and 4 °C indefinitely. Later, PCR amplifications were conducted in a 25 μL volume of Promega 5X Go Taq Green Buffer, 10 umol dNTPs mix, 100 U Promega Go Taq DNA Polymerase, 10 uM reverse primer and 10 uM forward primer, and 2 μL of cDNA. The thermal cycles were as follows: 3 min at 94 °C, followed by 35 cycles at 94 °C for 30 s, 56 °C for 45 s, and 72 °C for 1 min, with a final extension step of 72 °C for 7 min and 16 °C indefinitely. RT-PCR products were visualized in agarose electrophoresis gel and purified with DNA Clean & Concentrator TM-5 kit of ZYMO RESEARCH. Purified amplicons were directly submitted for Sanger sequencing in both directions using the same primers of PCR amplification.

## 3. Results

### 3.1. Identification of Viruses That Infect Galphimia spp.

**Figure 2:**
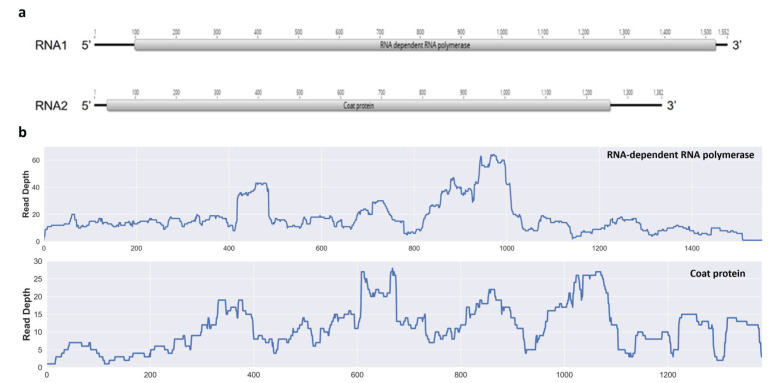
Proposed near-complete genome organization of Galphimia cryptic virus (GCV). (**a**), RNA molecules and corresponding genes are presented as gray boxes. (**b**), fold coverage of aligned reads for the two RNA sequences. Numbers represent nucleotide position in the virus genome.

Contigs generated from the HTS reads (i.e., >35,000,000 per sample) were compared against the GenBank database using BLASTx. As a result, two contigs displayed a significant top hit to a recently discovered partitivirus, the Vitis cryptic virus (GenBank accessions nos. OR474475 and OR474476); both sequences were obtained from a single plant belonging to the Morelos population. Based on sequence identity and the presence of typical domains/motifs, the proteins encoded by the putative virus were predicted to be RdRp and CP. These proteins shared the highest sequence identities with the Vitis cryptic virus (VCV), a member of the genus *Deltapartitivirus* within the family *Partitiviridae*. Thus, the near-complete genome of the novel virus (Figure 2a), named the Galphimia cryptic virus (GCV), comprises two segments with each containing a unique gene. The RNA1 of the GCV was determined to be 1552 nts long (GenBank accession no. OP168882.1) and shared a 65% identity (100% query coverage) with the VCV. Likewise, the GCV RNA2 (1382 nts; GenBank accession no. OP168883.1) displayed a homology of 44% (98% query coverage) with the VCV. Lastly, the mapping of the HTS reads identified 631 and 407 reads corresponding to RNA1 and RNA2, respectively, in both cases with 100% coverage (Figure 2b).

After comparing the GCV genes (i.e., amino acid sequence) with other deltapartiviruses from different plants via BLASTx, identity percentages greater than 60% for RdRp and around 40% for CP were obtained, as shown in Table 2. In both cases, greater similarities were found with the Dichroa partitivirus 2 and VCV. 

**Figure 3:**
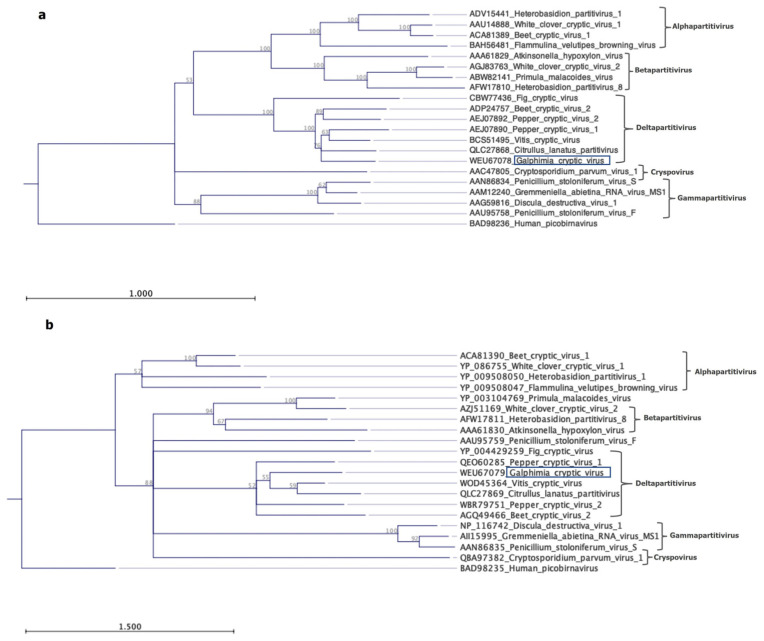
Phylogenetic trees showing the relationship of Galphimia cryptic virus with other members of the *Partitiviridae* family. The evolutionary history was inferred using amino acid sequence of the (**a**), RNA-dependent RNA polymerase and (**b**), coat protein. The human picobirna virus was included as outgroup. GenBank accession numbers are provided.

A phylogenetic analysis based on RdRp and CP sequences showed that the GCV clusters into the genus *Deltapartitivirus* along with other plant-infecting viruses (Figure 3a,b), supported by a bootstrap value above 90%. Overall, these results suggest a novel virus in the *Partitiviridae* family infecting *Galphimia* spp. 

### 3.2. Validation of Novel Virus by RT-PCR

The result of the HTS analysis was validated by end-point RT-PCR. As a result, amplicons with the expected size were generated from the “Morelos” plant, and bands of 503 and 565 bps corresponding to the RdRp and CP, respectively, were observed in the electrophoresis gel (Supplementary Figure S1). Lastly, Sanger sequencing allowed for the confirmation of the identities of the RdRp and CP genes from the GCV; a 100% homology with the original assembled sequences was obtained.

## 4. Discussion

Recently, molecular techniques, HTS, and bioinformatics algorithms have been used to discover new virus species associated with plant infections [17]. In this study, *Galphimia* spp. leaves, analyzed by HTS, revealed a new virus belonging to the *Partitiviridae* family named the Galphimia cryptic virus (GCV). Because the viral agent does not cause symptoms in the host plants, it was classified as a latent, a cryptic, or an asymptomatic virus [18]. When these types of virus-infected plants have developed the latency period, in which no viral replication occurs, usually there are low copy numbers of the viral particles and the viruses remain in a silenced status [9]. The read counts and coverage observed were consistent with lower levels of expression. 

In plants, partitivirus infections are transmitted vertically through seeds [19], and vertical transmission may occur in wild plants or crops. Seed infection provides the virus with suitable conditions to persist for long periods when the hosts and/or vectors are not available, because viral agents can survive within the seeds as long as it remains viable [20]. Viruses can reach the seeds by direct invasion of embryonic tissue and by infection of the ovules or pollen [21]. In several plant species, viral infection of the seeds has been proven, for example in *Polygonatum kingianum*, a high infection rate was found in the seeds by the Polygonatum kingianum cryptic virus 1 [19]. Other examples include the Raphanus sativus partitivirus 1 that infects *Raphanus sativus*; the Sinapis alba cryptic virus 1 that infects *Sinapis alba*; and the Brassica rapa cryptic virus 1 that infects *Brassica rapa* [22]. Considering that the plants used in this study were obtained from seeds of wild *Galphimia* spp., it is very likely that a GCV infection was acquired vertically from these seeds as well.

Latent viral infections can produce a mutualistic symbiosis between the virus and its host [23]. Artificial overexpression of the white clover cryptic virus CP in *Lotus japonicus* suppressed root nodulation, suggesting an increase in the endogenous abscisic acid (ABA) concentration and an activation of the plant’s innate immune response [24]. It has been studied that a CP of the cucumber mosaic virus, tobacco mosaic virus, and potato virus X contribute to pathogen resistance in *Arabidopsis thaliana* [25], *Capsicum annuum* [26], and *Solanum tuberosum* [27], respectively. Also, the RdRp of pepper severe mosaic virus activates a defense mechanism in *C. annuum* [28]. During viral pathogenesis, hormonal levels in plants improve the defense mechanism and provide resistance, but on several occasions, many plant viruses alter the hormone signaling [29]. In this sense, it has been studied that phytohormones such as auxins, brassinosteroids, cytokinins, and ABA are related to a plant’s defense and interactions with the virus and this action is correlated with levels of another phytoregulator such as ethylene, salicylic acid, and jasmonic acid [30]. Investigating the effect of the GCV on Galphimia’s immune response should be prioritized.

In the case of pathogenic viruses, viral infection affects the physiological and biochemical processes in the plant; this happens as a response to stressful conditions. The stresses induce changes at the cellular, physiological, and genome levels and the accumulation of secondary metabolites as the main forms of response in plants. It has been studied that grapevine red blotch virus increases the flavonoid and anthocyanin synthesis in *Vitis vinifera* [31], Telosma mosaic virus influences the production of phenols in *Passiflora edulis* [32], and saffron latent virus induce flavonoid synthesis in *Crocus sativus* [33]. A higher concentration of secondary metabolites can be good because they induce a resistance mechanism in the host plant, but on several occasions, it may produce an adverse effect such as reducing general growth and reproduction [29]. Whether or not a change in metabolites occurs in *Galphimia* spp. as result of a GCV infection is uncertain.

Finally, the effects that viruses produce on plants are directly correlated with variations in the environment where the plant has grown as well as the genetics of the hosts and viruses [34]. It should be considered that the “Morelos” population grows under different climatic conditions and it has been determined that it is genetically different from the “Guanajuato” population [35]. Taking into account the importance of secondary metabolism in the *Galphimia* genus and specifically the pathways related to the synthesis of terpenes, it would be useful to study the interactions between the GCV and the production of compounds of interest, which is the reason behind the importance of these plants.

## 5. Conclusions

A new virus was discovered in a symptomless *Galphimia* spp. plant by HTS and tentatively named the GCV. The virus was identified during a transcriptomic analysis of different populations of *Galphimia* spp. used to produce metabolites. Based on sequence identity and a phylogenetic analysis, the GCV represents a new member of the genus *Deltapartitivirus* (*Partitiviridae* family). The genome of the GCV is made up of two RNA molecules encoding for RdRp and CP. A missing task from this work is to complete the virus sequence via rapid amplification of the cDNA ends (RACE) method, and parts of the untranslated regions for both RNA molecules are lacking. Additionally, RT-PCR assays and Sanger sequencing were developed to confirm virus infection in the original source. The GCV is the first virus discovered in medicinal plants in Mexico. No symptoms were observed in the GVC-infected plant; however, potential effects of the GCV on the plant’s metabolism and the biology of the virus should be investigated.

## Figures and Tables

**Table 1:** Sequences of designed RT-PCR primers for Galphimia cryptic virus (GCV).

Gene Target	Primer Name	Primer Sequence	PCR Product Length (bp)
RNA-dependent RNA polymerase	GCV_RdRp	F 5′CATACACGCGCAATGTCTCG 3′ R 5′TGTGCCCAGTAAGTGTTCCC 3′	503
Coat protein	GCV_CP	F 5′TGTTTCGAACAGGGACTCCG 3′ R 5′TACTTCGTAACCACCGTGCC 3′	565

**Table 2:** Amino acid similarity between the two Galphimia cryptic virus (GCV) open reading frames to deltapartiviruses found in other host plants. Analysis conducted using BLASTx, and top ten results by percent identity are presented.

Virus Name	Host	Identity (%)
**RNA-dependent RNA polymerase (RdRp)**		
Vitis cryptic virus	*Vitis coignetiae*	65.40
Dichroa partitivirus 2	*Dichroa* sp.	64.54
Citrullus lanatus cryptic virus	*Citrullus lanatus*	63.83
Citrullus lanatus partitivirus	*Citrullus lanatus*	63.40
Polygonatum partitivirus 2	*Polygonatum kingianum*	62.00
Arceuthobium sichuanense virus 7	*Arceuthobium sichuanense*	60.76
Mercurialis partitivirus 1	*Mercurialis perennis*	62.13
Raphanus sativus cryptic virus 3	*Raphanus sativus*	60.93
Panax cryptic virus 1	*Panax* sp.	61.91
Coriandrum sativum deltapartitivirus 2	*Coriandrum sativum*	61.36
**Coat protein (CP)**		
Vitis cryptic virus	*Vitis coignetiae*	45.21
Dichroa partitivirus 2	*Dichroa* sp.	45.64
Arceuthobium sichuanense virus 7	*Arceuthobium sichuanense*	41.69
Pittosporum cryptic virus 1	*Pittosporum tobira*	42.65
Dactylorhiza cryptic virus 3	*Dactylorhiza* sp.	39.36
Panax cryptic virus 1	*Panax* sp.	39.61
Polygonatum partitivirus 2	*Polygonatum kingianum*	40.15
Citrullus lanatus partitivirus	*Citrullus lanatus*	40.21
Citrullus lanatus cryptic virus	*Citrullus lanatus*	40.57
Coriandrum sativum deltapartitivirus 2	*Coriandrum sativum*	39.48

## Data Availability

The data obtained in this study were submitted to the GenBank database under the accession numbers OP168882 and OP168883.

## Supplementary Materials

The following are available online at https://www.mdpi.com/article/10.3390/pathogens13060504/s1, Figure S1. RT-PCR testing to confirm the presence of Galphimia cryptic virus (GCV) in Morelos population.
