# Supplementary material for: A Novel Cryptic Virus Isolated from Galphimia spp. in Mexico
Source: Pathogens. 2024 Jun 13;13(6):504. doi: 10.3390/pathogens13060504 (PMC11207071; doi:10.3390/pathogens13060504)
Supplement: Supplementary file 1 [file pathogens-13-00504-s001.zip › pathogens-3008454-supplementary.pdf]

A novel cryptic virus isolated from Galphimia spp. in Mexico

Dianella Iglesias, Kristian Stevens, Ashutosh Sharma and Alfredo Diaz-Lara

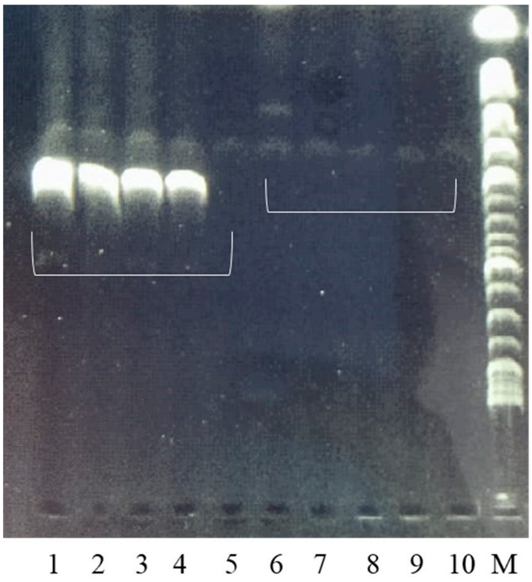

Supplementary Figure S1. RT-PCR testing to confirm the presence of Galphimia cryptic virus (GCV) in Morelos population. T2 sample with several replicates. 1-6, RT-PCR targeting the RdRp of GCV. 7-10, RT-PCR targeting the CP. M, molecular weight market.
